# Visualizing the Prostate Gland by MR Imaging in Young and Old Mice

**DOI:** 10.1371/journal.pone.0055746

**Published:** 2013-03-01

**Authors:** Murali Ravoori, Jyoti Duggal, Mihai Gagea, Lin Han, Sheela Singh, Ping Liu, Wei Wei, Dustin K. Ragan, James A. Bankson, Jingfei Ma, Vikas Kundra

**Affiliations:** 1 Department of Experimental Diagnostic Imaging, The University of Texas MD Anderson Cancer Center, Houston, Texas, United States of America; 2 Department of Veterinary Medicine and Surgery, (Section of Body Imaging), The University of Texas MD Anderson Cancer Center, Houston, Texas, United States of America; 3 Department of Biostatistics, The University of Texas MD Anderson Cancer Center, Houston, Texas, United States of America; 4 Department of Imaging Physics, The University of Texas MD Anderson Cancer Center, Houston, Texas, United States of America; 5 Department of Diagnostic Radiology, The University of Texas MD Anderson Cancer Center, Houston, Texas, United States of America; Queensland University of Technology, Australia

## Abstract

**Purpose:**

Prostate imaging requires optimization in young and old mouse models. We tested which MR sequences and field strengths best depict the prostate gland in young and old mice; and, whether prostate MR signal, size, and architecture change with age.

**Technique:**

Magnetic resonance imaging (MRI) of the prostate of young (2 months) and old (18 months) male nude mice (n = 6) was performed at 4.7 and 7 T and SCID mice (n = 6) at 7 T field strengths, using T1, fat suppressed T1, DWI, T2, fat suppressed T2, as well as T2-based- and proton density-based Dixon “water only” sequences. Images were ranked for best overall sequence for prostate visualization, prostate delineation, and quality of fat suppression. Prostate volume and signal characteristics were compared and histology was performed.

**Results:**

T2-based-Dixon “water only” images ranked best overall for prostate visualization and delineation as well as fat suppression (n = 6, P<0.001) at both 4.7 T and 7 T in nude and 7T in SCID mice. Evaluated in nude mice, T2-based Dixon “water only” had greater prostate CNR and lower fat SNR at 7 T than 4.7 T (P<0.001). Prostate volume was less in older than younger mice (n = 6, P<0.02 nude mice; n = 6, P<0.002 SCID mice). Prostate T2 FSE as well as proton density-based and T2-based-Dixon “water only” signal intensity was higher in younger than older mice (P<0.001 nude mice; P<0.01 SCID mice) both at 4.7 and 7 T. This corresponded to an increase in glandular hyperplasia in older mice by histology (P<0.01, n = 6).

**Conclusion:**

T2-based Dixon “water only” images best depict the mouse prostate in young and old nude mice at 4.7 and 7 T. The mouse prostate decreases in size with age. The decrease in T2 and T2-based Dixon “water only” signal with age corresponds with glandular hyperplasia. Findings suggest age should be an important determinant when choosing models of prostate biology and disease.

## Introduction

Prostate research has been limited by a dearth of methods for visualizing the gland in animal models [1]–[5]. MRI has been used, but has relied primarily on non-fat suppressed T2-weighted imaging, generally to visualize large prostate tumors [3], [5]–[8]. However, multiple other sequences have not been compared for visualizing the prostate. Commonly, T1 and T2-weighted sequences are used to delineate organs, and diffusion-weighted imaging (DWI) is now commonly used clinically. Newer sequences such as Dixon [9] -based sequences can be applied to obtain “water” weighting or “fat” weighting; the degree of weighting is based on the original sequence weighting such as T2 or proton density.

Compared to the other modalities, the spatial resolution and contrast of MR enables exquisite anatomic depiction. However, both image contrast and spatial resolution are influenced by field strength. Currently, 4.7T and 7T magnets are most commonly used for small animal imaging. Clinically, higher field strength has not always proved advantageous, especially for body imaging [10].

When mice are utilized as models of prostate biology and disease, they are most often used at a young age of approximately 8–12 weeks. In humans, the prostate changes with age. For example, the prostate tends to increase in size from youth to young adult. In older adults, the prostate commonly increases in size secondary to benign prostatic hyperplasia. Epidemiologically, prostate cancer is more commonly found in older men. Comparison of MR imaging characteristics of the prostate in young and old mice has not been performed. We chose nude and SCID mice in the current study, since these are commonly used models.

In this study, we tested multiple MR sequences and field strengths to determine which best depicts the prostate gland in young and old mice; and, assessed whether age alters mouse prostate MR signal, size and architecture in young versus old mice.

## Materials and Methods

### Animal Experiments

The animals were cared for in accordance with guidelines set forth by the Association for Assessment and Accreditation of Laboratory Animal Care and the U.S. Public Health Service Policy on Humane Care and Use of Laboratory Animals. All animal experiments were approved by the Institutional Animal Care and Use Committee of The University of Texas M. D. Anderson Cancer Center. Magnetic resonance imaging (MRI) of the prostate gland of young (2 months) and old (18 months) male nude mice (n = 6) was performed at 4.7 and 7 T and SCID mice (n = 6) at 7 T field strengths.

### Magnetic Resonance Imaging Protocol

For all MR imaging experiments, animals were anesthetized with 2% isoflurane. Phantoms were imaged with the animal. Imaging was done in 4.7 and 7 T small animal MR scanners (Biospec, Bruker Biospin MRI, Inc., Billerica, MA) using transmit/receive volume coils with 35 mm inner diameter. Seven different MRI sequences were used for both 4.7 and 7 T field strengths. The following imaging sequences were used to acquire images in the axial plane at 4.7 T; except as noted, a field-of-view of 4×3 cm^2^, a matrix of 256×192, and spatial resolution of 156 micrometers are used: a T2-weighted Fast spin echo (FSE) acquisition with fat saturation (T2-FS, echo time, 80 ms; echo train length, 12; receiver bandwidth, 101 kHz; repetition time, 3159 ms; nex, 4; slice thickness, 1 mm), T2-weighted FSE acquisition with Dixon fat/water separation (echo time, 60 ms; echo train length, 8; repetition time, 3700 ms; nex, 2; slice thickness, 0.5 mm), FSE proton density (PD) weighted Dixon (echo time, 7 ms; echo train length, 8; repetition time, 3000 ms; nex, 2; slice thickness, 0.5 mm), respiratory-gated diffusion weighted imaging (DWI, echo time, 33 ms; B value of 300 s/mm^2^; repetition time approximately 2 s, determined by respiratory rate; nex, 1; slice thickness, 1 mm; matrix, 128×128; spatial resolution, 312 micrometers), T1 MSME (echo time, 8.4 ms; repetition time, 900 ms; nex, 1; slice thickness, 1 mm), T1 with fat saturation (T1-FS, echo time, 8.4 ms; repetition time, 900 ms; nex, 1; slice thickness, 1 mm; spatial resolution, 195 micrometers), and T2 without fat suppression (echo time, 80 ms; repetition time, 3031 ms; nex, 4; slice thickness, 1 mm).

The following imaging sequences were also to acquire images in the axial plane at 7 T; except as noted, a field-of-view of 4×3 cm^2^, a matrix of 256×192, and spatial resolution of 156 micrometers are used: a T2-weighted Fast spin echo (FSE) acquisition with fat saturation (T2-FS, echo time, 65 ms; echo train length, 12; receiver bandwidth, 101 kHz; repetition time, 4000 ms; nex, 3; slice thickness, .75 mm), T2-weighted FSE acquisition with Dixon fat/water separation (echo time, 60 ms; echo train length, 8; repetition time, 3700 ms; nex, 2; slice thickness, 0.5 mm), FSE proton density (PD) weighted Dixon (echo time, 7 ms; echo train length, 8; repetition time, 3000 ms; nex, 2; slice thickness, 0. 5 mm), respiratory-gated diffusion weighted imaging (DWI, echo time, 33 ms; B value of 300 s/mm^2^; repetition time approximately 2 s, determined by respiratory rate; nex, 1; slice thickness, 1 mm; matrix, 128×128; spatial resolution, 312 µm), T1 MSME (echo time, 7.8 ms; repetition time, 500 ms; nex, 2; slice thickness, .75 mm), T1 with fat saturation (T1-FS, echo time, 7.8 ms; repetition time, 500 ms; nex, 2; slice thickness, .75 mm; field of view 4×3 cm; matrix, 256×192; spatial resolution, 156 micrometers), and T2 without fat suppression (echo time, 80 ms; repetition time, 4000 ms; nex, 3; slice thickness, .75 mm). The nude mice (n = 6 for young, 2 months old; n = 6 for old, 18 months old) were imaged using both 4.7 and 7 T field strengths while the SCID mice (n = 6 for young, 2 months old; n = 6 for old, 18 months old) were imaged only at 7 T.

For Dixon related sequence image acquisition, we modified the conventional fast spin echo pulse sequence to acquire two interleaved input images (one in-phase and the other 180° opposed-phase) [11], [12]. The 180° phase shift was achieved by increasing the echo spacing (the time interval between two successive 180° RF refocusing pulses in the echo train) and by shifting the readout gradients/data acquisition window from the conventional spin echo location by approximately 750 microseconds at 4.7T and 504 microseconds at 7T. After data acquisition, we used a previously-published phase correction algorithm to generate water-only and fat-only images for each slice [13]. In contrast to other-known three-point Dixon processing algorithms [14]–[16] the phase correction algorithm we used requires only two images (one with the water and fat in-phase and the other with the two signals 180° opposed-phase) and circumvents the need for using error-prone direct phase unwrapping. Instead, the algorithm uses a fully automated region growing process to determine the phase vector distribution, which has been shown to be sufficient for water and fat separation [13].

### Interpretation of MR Images

All of the MRI sequence images were arranged randomly and blinded interpretation was done independently by a Board-certified radiologist (reader 1) and a scientist with more than five years experience in small animal imaging (reader 2) to rank the quality of prostate gland visualization, border delineation, and fat suppression. The images were graded on a scale of 1 to 5 with 1 = not seen, 2 = poor, 3 = fair, 4 = good, and 5 = excellent. In addition, the images for each mouse were ranked as overall best MR sequence from 1 to 14 in the nude mice including 4.7T and 7T and 1 to 7 in the SCID mice at 7T with 1 being the best image.

### Prostate Gland Measurements

The periphery of the prostate on the 7T MRI T2-based Dixon “water only” images was manually traced on axial images using a region of interest (ROI), and the area of the enclosed region was calculated. Prostate gland volume measurements were performed as previously described [17] using Image J software (National Institutes of Health, Bethesda, MD) and signal intensity ratio was measured by placing a region of interest (ROI) around the entire prostate gland in a central slice of T2-FS and T2-based Dixon “water only” images and dividing it by signal intensity of the standard water phantom used in each acquisition. Signal to noise ratio of periprostatic fat was measured by manually placing a ROI in fat adjacent to the prostate gland in a central slice of T2FSE, T2-FS, PD-based Dixon “water Only” and T2-based Dixon “water only” images. This was divided by the reference signal in the water phantom. Contrast to noise ratio of the prostate was measured by subtracting the fat signal from the signal in the prostate and dividing the difference by the signal in the reference water phantom.

### Histological Examination

The prostates of 6 young and 6 old Nude mice and of 6 young and 6 old SCID mice were examined microscopically. After euthanasia, the animals were necropsied and the prostate glands were collected together with the urogenital tract and placed on a piece of index card before immersion in 10% neutral buffered formalin in order to preserve the anatomic orientation of tissues after fixation [18]. After 48 hours of fixation in formalin, the dorsal, lateral and ventral lobes of each prostate were sectioned in transversal (dorso-ventral) plane at 2 mm interval, and the anterior lobe (coagulating gland) was oriented in a longitudinal plane in tissue cassettes for histologic processing. From paraffin-embedded tissues, sections of 4 µm thickness were mounted on glass slides and stained with hematoxylin and eosin (H&E). A veterinary pathologist examined microscopically the H&E stained sections. The hyperplastic changes of the epithelium of prostate glands were evaluated and graded with a score from 0 to 4 as follows: 0 =  normal (no hyperplastic changes), 1 =  minimal hyperplasia, 2 =  mild, 3 =  moderate, and 4 =  marked hyperplasia. Presence of any other histologic change of the prostate was also recorded.

### Statistical Analysis

For the calculations of prostate gland volume, signal intensity, 4.7T vs 7T T2-based Dixon “water only” ranking, and pairwise comparisons of T2 Dixon “water only” vs T2 FS groups were compared using two-sided *t*-tests. Analyses were performed using Microsoft Excel 2007 (Microsoft Corp., Redmond, WA) and for all results, P<0.05 was considered statistically significant.

For the blinded interpretation of all the MR sequences, a multiple comparison using the Tukey-Kramer method was used to compare the overall ranking, the quality of visualization, delineation, and fat suppression across all groups for each of the imaging modalities (4.7T vs 7T) and for young vs old mice. A P-value of ≤0.05 was considered statistically significant.

## Results

### Prostate Depiction

**Figure 1:**
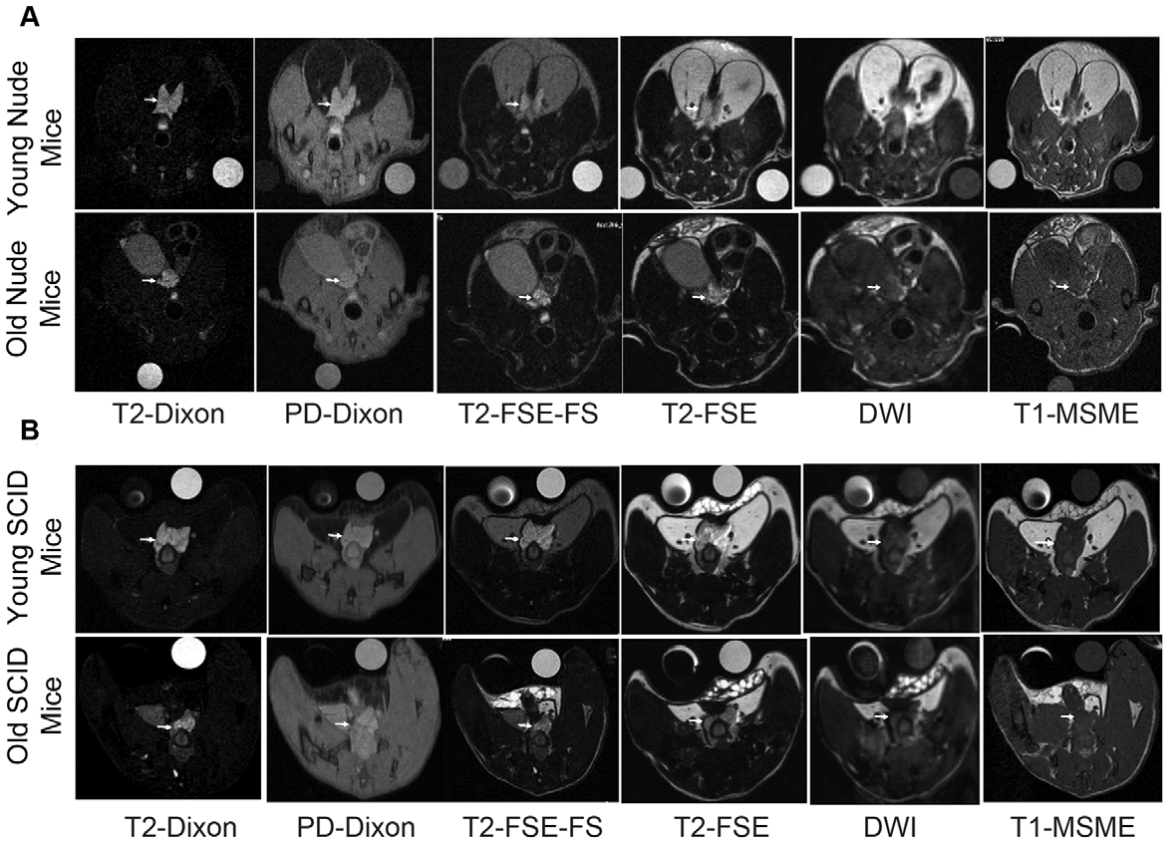
Representative MR images of the prostate gland of nude (4.7T, A), SCID (7T, B) young (top) and old (bottom) mice with different MR sequences. Arrow indicates the prostate gland.

**Figure 2:**
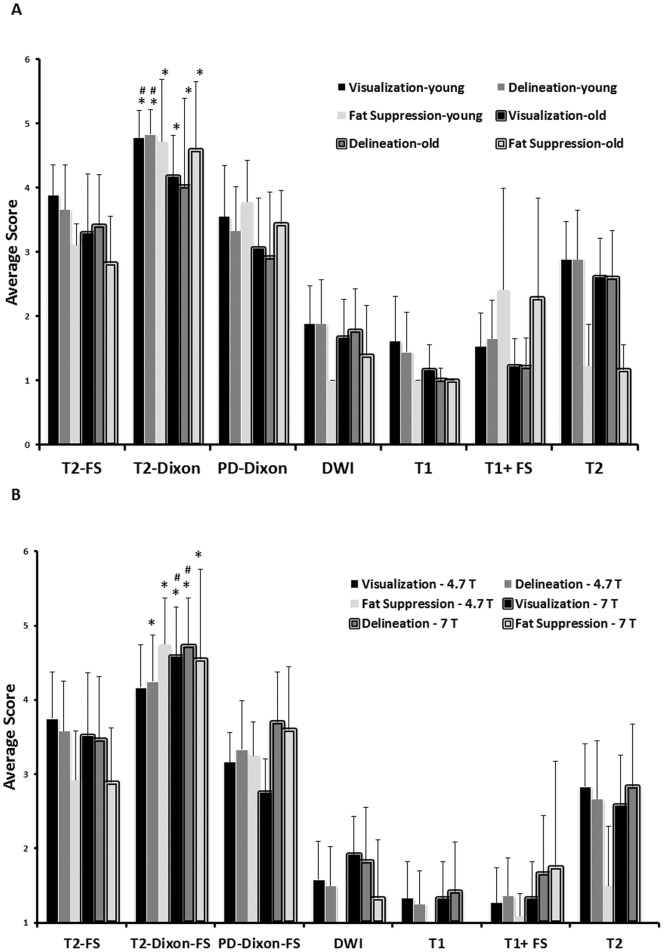
Prostate visualization, prostate border delineation, and fat suppression of young and old SCID and nude mice ranked significantly higher on T2-Dixon “water only” overall and generally better than T2 FS images using either 4.7 T or 7T scanners. A) 7T magnet, young vs old mice (*, P<0.01, T2-Dixon vs T2 FS; #, P<0.001, T2-Dixon “water only” young vs old); B) 4.7 T vs 7T (*, P<0.01, T2-Dixon vs T2 FS; #, P<.05, T2-Dixon “water only” 4.7T vs 7T). Reader 1 data presented.

Figure 1 (A & B) demonstrates representative images of the prostate gland. Note that fat suppressed type images separate the prostate gland from surrounding fatty tissue and the quality of fat suppression is best with the T2-based Dixon “water only” images. The T2-based images appear superior to proton density based Dixon images due to less background signal. Note also that the prostate has high signal on T2-weighted images and low signal intensity on T1-weighted images, consistent with high water content of this organ. T2-based Dixon “water only” sequence ranked the highest by both readers in overall image quality for both nude and SCID mice as compared to the other MR imaging sequences at both 4.7T and 7T (Table 1). Images acquired at 7T using the T2-based Dixon “water only” ranked higher than similar 4.7T images in nude mice (P<.038, n = 6) for overall best sequence. For both young and old mice, T2-Dixon “water only” performed better overall than other sequences for prostate visualization, margin delineation, and fat suppression at 4.7T and 7T by both readers, and specifically better than T2 FS excluding visualization at 4.7T for the first reader (Table 2, Figure 2A and B) but including visualization at 4.7T for the second reader. For young or old mice, fat signal to noise ratio (SNR) was lower in T2-Dixon “water only” than T2 FS at 7T (P<.05, n = 12), and in old mice at 4.7T (P<.01, n = 6); furthermore, prostate contrast to noise ratio (CNR) was higher at both 7T (P<.05, n = 12) and 4.7T (P<.05, n = 6) in young or old mice (Table 3). Individually comparing the highest rated sequences, fat signal to noise ratio (SNR) was also lower in T2-Dixon “water only” than PD “water only” or T2 at 4.7T and 7T (P<.001, n = 12 and 24); moreover, prostate contrast to noise ratio (CNR) was higher than PD “water only” at 7T (P<.01, n = 24) and T2 at 4.7T and 7T (P<.001, n = 12 and 24). Prostate visualization and margin delineation were better among young mice than old mice by linear mixed model analysis by the first reader and second reader other than the poorly performing DWI and T1 with contrast and within T2 “water only images” by the first reader (Figure 2A) and second reader; in addition, fat suppression was also rated superior by the second reader (P<.05). Prostate visualization and margin delineation were better at 7T than 4.7T by linear mixed model analysis by reader 1 only and within T2 “water only images” by reader 1 (Figure 2B) only. Fat signal to noise ratio (SNR) was lower at 7T than 4.7T (P<.001, n = 24, 12 respectively); furthermore, prostate contrast to noise ratio (CNR) was higher at 7T than 4.7T (P<.001, n = 24, 12 respectively, Table 4).

**Table 1:** Comparison of overall best MR sequence at 4.7T and 7T.

Strain, Field Strength	Overall Best Sequence	Overall P-value
Nude, 4.7T	T2-Dixon>T2-FS>PD-Dixon>T2RARE> DWI>T1+FS>T1*	<0.001
Nude, 7T	T2-Dixon>PD-Dixon>T2-FS>T2RARE> DWI>T1+FS>T1	<0.001
SCID, 7T	T2-Dixon>T2-FS>T2RARE>PD-Dixon> DWI>T1>T1+FS	<0.001

* Reader 2 rated PD-Dixon>T2FS for Nude 4.7T and equivalent for nude or SCID mice at 7T.

**Table 2:** Comparison of visualization, border delineation, and fat suppression at 4.7T and 7T (P-values).

Strain, Field Strength	Sequence	Visualization	Delineation	Fat Suppression
Nude, 4.7T	T2-Dixon “water only” vs all other	*P<0.001	P<0.001	P<0.001
Nude and SCID 7T	T2-Dixon “water only” vs all other	P<0.001	P<0.001	P<0.001

* Overall P-values.

**Table 3:** T2-Dixon “water only” vs T2-FS Fat SNR and Prostate CNR.

FAT SNR	Field Strength	Mouse age	P-value
T2-Dixon “water only” lower	7T	Young Old	<0.05 <0.05
T2-Dixon “water only” lower	4.7T	Young Old	<0.01 >0.05
**Prostate CNR**			
T2-Dixon “water only” higher	7T	Young Old	<0.05 <0.05
T2-Dixon “water only” higher	4.7T	Young Old	<0.05 <0.05

**Table 4:** 7T vs 4.7T T2-Dixon “water only” Fat SNR and Prostate CNR.

	P-value
7T Fat SNR lower	<0.001
7T Prostate CNR higher	<0.001

### Prostate Gland Measurements

**Figure 3:**
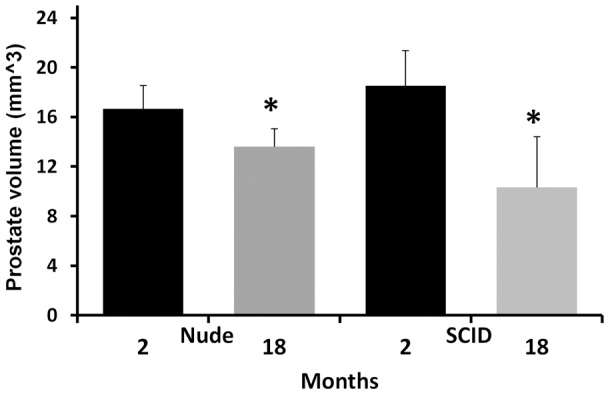
Prostate gland volume derived from 7T MR images of young and old SCID and nude mice (*, P<0.02, n = 6 for young vs old nude mice; P<0.002, n = 6 for young vs old SCID mice).

**Figure 4:**
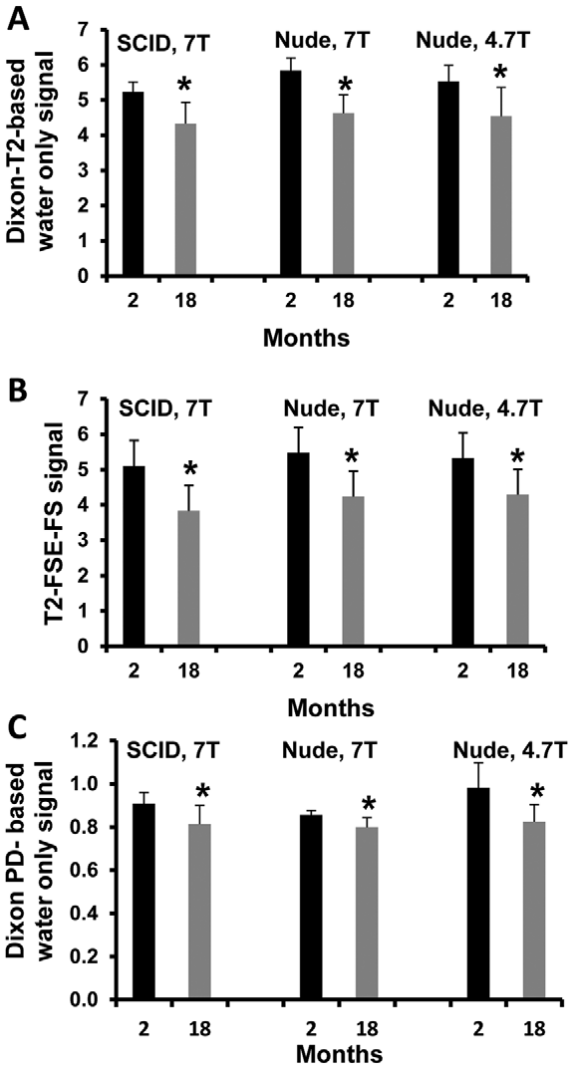
Prostate gland signal intensity measured using T2-Dixon-“water only” (A), T2 FS (B), or (C) PD-Dixon-“water only” sequences in young and old SCID and nude mice both at 4.7 T and 7T (*, P<0.05, n = 6).

The volume of the prostate glands decreased significantly in older mice as compared to younger mice (P<0.02, n = 6 for nude mice; P<0.002, n = 6 for SCID, Fig. 3, measured at 7T). The prostate gland signal intensity on T2-based Dixon “water only” and on T2 FS decreased significantly in old mice compared to young mice (T2-based Dixon “water only”, P<0.05, n = 6 for nude or SCID mice at 7T and P<0.05, n = 6 for nude mice at 4.7T, Fig. 4A; T2 FS, P<0.02, n = 6 for nude mice at 4.7T and P<0.05, n = 6 for nude mice at 7T; P<0.05, n = 6 for SCID mice at 7T, Fig. 4B), suggesting a more hydrated gland in younger mice. Similar findings were noted with the proton density-based Dixon “water only” sequence.

### Histological Analysis

**Figure 5:**
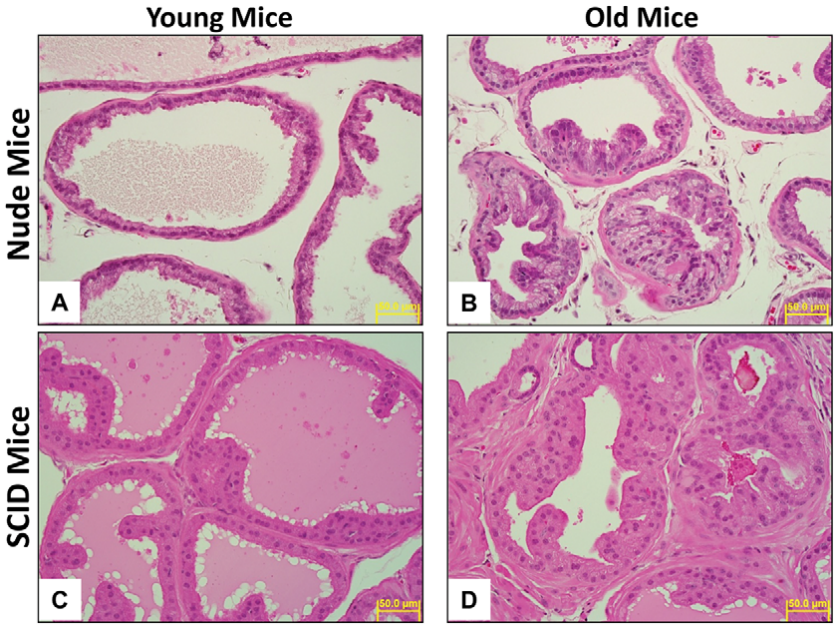
Prostate gland histology of young and old mice, H&E stain, ×400. A) Nude young mice, minimal hyperplasia, grade 1; B) Nude old mice, mild hyperplasia, grade 2; C) SCID young mice, minimal hyperplasia, grade 1; D) SCID old mice, moderate hyperplasia, grade 3.

Histologic examination of the mice demonstrated that the degree of glandular hyperplasia of the prostate gland increased in older mice in comparison with younger mice in both nude (P≤0.003, Table 5, Fig. 5A & B) and SCID mice (P≤0.007, Table 5, Fig. 5C & D). Young nude mice had normal or only minimal hyperplasia of the prostate gland epithelium (an average score of 0.67 on a scale of 0 to 4, Table 5, Fig. 5A) in comparison with old nude mice that commonly had mild or moderate hyperplasia (an average score of 2.0, Table 5, Fig. 5B). A similar pattern of changes was observed in the SCID mice (Table 5, Fig. 5C & D). No neoplastic lesions were observed in any of the mice of this study. Visually, younger mice had more proteinaceous secretion within the lumen of prostate glands in comparison with older mice in both nude and SCID mice.

**Table 5:** Average grade of hyperplasia of prostate gland epithelium in young and old SCID and nude mice.

Strain	Young Mice	Old Mice	P-value
Nude	0.67	2.00	<0.003, n = 6
SCID	1.33	2.67	<0.007, n = 6

## Discussion

Mouse models of prostate cancer have tended to involve young mice approximately 8–12 weeks old. This investigation demonstrated that there are significant imaging and histological differences between young and older mice. This suggests that the age of the animal should be taken into account when selecting animal models of prostate biology and disease, and when analyzing prostate images. Older mice had decreased prostate size compared to younger animals in both nude and SCID mice. This is in contradistinction to humans in whom prostate size tends to increase with age, commonly due to benign prostatic hyperplasia (BPH). In humans, the increased size due to BPH can lead to obstructive symptoms and imaging findings such as an enlarged central gland (the anatomic transition and central zones) with heterogeneous but primarily low T1 and T2 signal [19]. With long standing obstruction, bladder trabeculation can be seen in humans. In comparison, the peripheral zone in humans tends to have low T1 and high T2 signal [19].

In mice, T2-weighted fat suppression signal and T2-based Dixon “water only” signal decreased with age. The mouse prostate does not have the same lobes as in humans. A human prostate has peripheral, central, and transitional zones. The prostate gland in mice consists of three lobes around the urethra (ventral, lateral and dorsal), and one lobe along the lesser curvature of each seminal vesicle named anterior lobe or coagulating gland. The histology of each lobe is distinct [20]. Due to the anatomic and histologic differences, a similar correlate to BPH in terms of zonal changes may not be expected. However, within the mouse prostate, we did find prostate gland hyperplasia in older mice. We suggest that this corresponds to the decreased T2-weighted fat saturation signal and T2-based Dixon “water only” signal, as well as decreased size since the hyperplastic glandular epithelium may consist of less hydrated tissue, may be functionally less active, and may take up luminal space that would normally be filled by prostatic secretions in younger mice. T2-weighted fat saturation signal and T2-based Dixon “water only” signal primarily reflect the degree of hydration of tissue. The proton density “water only” findings also suggest the less hydration with age.

Multiple sequences for imaging the prostate have not been compared in mouse models. Most MR imaging of the prostate has used T2-weighting without fat saturation. We chose nude and SCID mice as models to be able to assess consistency of results in two different models and because these two models are commonly used in studies of prostate pathology, for example, in orthotopic models of prostate cancers. We hypothesized that due to its glandular nature, the prostate would be better seen using sequences that evaluate the degree of hydration. Indeed T2 and “water only” based sequences ranked high in terms of prostate visualization. We also hypothesized that because the prostate is surrounded by fat, sequences that reduced fat signal would better visualize the gland; and, indeed, these ranked higher for margin delineation. The findings were supported objectively by the fat SNR and prostate CNR data. T2-weighted images without fat suppression did not depict the prostate as well as sequences that also included fat suppression techniques. Among the sequences, Dixon based sequences fared well. This is the first application of this technique for mouse prostate imaging.

Dixon based techniques use frequency shift to separate out “water” and “fat” signal. This post-processing technique can be applied to sequences that exploit different contrast mechanisms. The primary sequence is important as demonstrated by the different appearances of the “water only” images generated from primary T2-weighted versus proton density-weighted images. The contrast on the “water only” images from the primary proton density-weighted sequence was lower than that from the primary T2-weighted sequence. The latter resulted in a high signal (bright) prostate which contrasted with the low signal (dark) fat. The T2-weighted “water only” Dixon sequence was robust since it was ranked highest among the different mouse genetic backgrounds, among young and old mice, and among MR magnets of different field strengths. Findings were supported objectively by the fat SNR and prostate CNR data. Both readers ranked T2-weighted “water only” Dixon sequence as the best overall sequence.

Although higher field strength should result in higher spatial resolution, it can also result in decreased soft tissue contrast. Indeed, in some body applications in humans, higher 3T field strength has not always shown superiority to lower 1.5T imaging [21]–[23]. In the current study, T2 weighted “water only” Dixon images in nude mice ranked higher for overall best sequence at 7T than at 4.7T by reader 1. Findings were supported objectively by the fat SNR and prostate CNR data.

This is the first study to directly compare multiple MR sequences for imaging of the prostate gland in mouse models. The results support the use of T2-Dixon-“water only” imaging at both 4.7 and 7 T magnetic field strengths in young and old mice for imaging the prostate gland. They also demonstrate that the mouse prostate has less T2 and T2-based “water only” signal suggesting less hydration, but unlike humans, the mouse prostate decreases in size with age; mechanistically, this is likely related to the mouse prostate undergoing glandular hyperplasia. Thus, the age of the animal and MR sequence chosen are critical parameters when choosing mouse models for studying the prostate gland.
